# Supplementary material for: Dynalign II: common secondary structure prediction for RNA homologs with domain insertions
Source: Nucleic Acids Res. 2014 Nov 21;42(22):13939–48. doi: 10.1093/nar/gku1172 (PMC4267632; doi:10.1093/nar/gku1172)
Supplement: SUPPLEMENTARY DATA [file supp_gku1172_nar-02021-z-2014-File012.zip › manual/Text/DynalignDotPlot.html]

RNAstructure Command Line Help: DynalignDotPlot


|  |  |  |
| --- | --- | --- |
|  | RNAstructure Command Line Help DynalignDotPlot | - Contents - Index |
| DynalignDotPlot is used to create a pairing energy dot plot for the structures of one of the two sequences folded by a Dynalign calculation. USAGE: DynalignDotPlot <save file> <ps file> [options] Required parameters: <save file> \| The name of a binary Dynalign save file. These binary save files usually end in ".dsv", and contain probability data from a previous Dynalign calculation.; <output file> \| The name of a file to which output will be written. Options which don't require added values: -h, -H, --help \| Display the usage details message.; --s2, -S2, --sequence2 \| Use sequence 2, rather than the default of sequence 1.; --svg \| Specify that the output file should be an SVG image file, rather than a Postscript image file.; -t, -T, --text \| Write dot plot as a text file, rather than a Postscript image file. Options which require added values: -e, -E, --entries \| Specify the number of categories in the plot legend. Default is 5 entries.; -max, -MAX, --maximum \| Specify the maximum value in the plot in kcal/mol. Default is the largest valid value in the plot.; -min, -MIN, --minimum \| Specify the minimum value in the plot in kcal/mol. Default is the smallest valid value in the plot. References: 1. Reuter, J.S. and Mathews, D.H. "RNAstructure: software for RNA secondary structure prediction and analysis." *BMC Bioinformatics*, 11:129. (2010). 2. Mathews, D. H. "Predicting a set of minimal free energy RNA secondary structures common to two sequences." *Bioinformatics*, 21:2246-2253. (2005). | | |
| Visit The Mathews Lab RNAstructure Page for updates and latest information. | | |
